# Mechanisms of GnRH-Induced Extracellular Signal-Regulated Kinase Nuclear Localization

**DOI:** 10.1371/journal.pone.0040077

**Published:** 2012-07-12

**Authors:** Christopher J. Caunt, Rebecca M. Perett, Robert C. Fowkes, Craig A. McArdle

**Affiliations:** 1 Department of Biology and Biochemistry, University of Bath, Bath, United Kingdom; 2 School of Clinical Sciences, University of Bristol, Bristol, United Kingdom; 3 Endocrine Signaling Group, Royal Veterinary College, London, United Kingdom; University of Sassari, Italy

## Abstract

Gonadotropin-releasing hormone receptors (GnRHR) mediate activation and nuclear translocation of the extracellular signal regulated kinases 1 and 2 (ERK) by phosphorylation on the TEY motif. This is necessary for GnRH to initiate transcriptional programmes controlling fertility, but mechanisms that govern ERK targeting are unclear. Using automated microscopy to explore ERK regulation in single cells, we find that GnRHR activation induces marked redistribution of ERK to the nucleus and that this effect can be uncoupled from the level of TEY phosphorylation of ERK. Thus, 5 min stimulation with 100 nM GnRH increased phospho-ERK levels (from 89±34 to 555±45 arbitrary fluorescence units) and increased the nuclear:cytoplasmic (N:C) ERK ratio (from 1.36±0.06 to 2.16±0.05) in the whole cell population, but it also significantly increased N:C ERK in cells binned according to phospho-ERK levels. This phosphorylation unattributable component of the ERK translocation response occurs at a broad range of GnRHR expression levels, in the presence of tyrosine phosphatase and protein synthesis inhibitors, and in ERK mutants unable to undergo catalytic activation. It also occurred in mutants incapable of binding the DEF (docking site for ERK, F/Y-X-F/Y-P) domains found in many ERK binding partners. It was however, reduced by MEK or PKC inhibition and by mutations preventing TEY phosphorylation or that abrogate ERK binding to D (docking) domain partners. We therefore show that TEY phosphorylation of ERK is necessary, but not sufficient for the full nuclear localization response. We further show that this “phosphorylation unattributable” component of GnRH-mediated ERK nuclear translocation requires both PKC activity and association with partner proteins via the D-domain.

## Introduction

The gonadotropin-releasing hormone (GnRH) is a hypothalamic decapeptide (pGlu-His-Trp-Ser-Tyr-Gly-Leu-Arg-Pro-Gly-NH_2_) that is the master control hormone in reproduction [1]. GnRH is secreted in a pulsatile fashion by the hypothalamus and acts on G_q/11_-coupled seven transmembrane (7TM) GnRH receptors (GnRHRs) in gonadotrope cells of the pituitary. This causes the synthesis and secretion of luteinizing hormone (LH) and follicle-stimulating hormone (FSH). GnRHR activation initiates several intracellular signalling cascades in gonadotropes, but activation of the ERK extracellular signal-regulated kinase) MAPK (mitogen-activated protein kinase) cascade is responsible for a large proportion of the biological effects elicited by GnRH [1]–[3]. For example, ERK-dependent transcription of the early growth response gene-1 (Egr-1) transcription factor is required for LH transcription, and female mice lacking ERK in the pituitary fail to ovulate [4].

GnRH can cause ERK cascade activation through a variety of signalling routes, such as activation of protein kinase C (PKC) isozymes, and/or transactivation of the epidermal growth factor receptor (EGFR). The specific route appears to be dependent upon cellular context, but studies to date indicate they converge at the level of Raf kinase activation [2], [3]. Activated Raf can then phosphorylate and activate the cytosolic kinases, MEK (MAPK/ERK kinase) 1 and 2, which, in turn, phosphorylate ERKs 1 and 2 (herein specific ERKs are referred to as ERK1 or ERK2 and the term ERK is used to mean ERK1 and/or ERK2) on Thr and Tyr residues of a TEY activation motif [5]–[7]. This typically causes dissociation from a number of cytoplasmic anchors (including MEK), resulting in nuclear accumulation of ERK [8], [9]. This relocalization of ERK represents a key event in the transmission of extracellular signals to the nucleus, as it is essential for ERK to phosphorylate nuclear substrates involved in altering gene expression [10]. Appropriate regulation of ERK nuclear targeting is therefore essential during execution of cell fate decisions, but the mechanisms controlling it remain incompletely understood.

ERK contains no recognizable nuclear localization or export signals and movement across the nuclear envelope can occur via energy dependent and independent routes [11]–[13]. ERK shuttling to and from the nucleus is also very rapid, suggesting that nucleo-cytoplasmic ERK distribution is chiefly governed by the availability of ERK binding sites in the nucleus or cytoplasm [14], [15]. Rates of shuttling can be rapidly modulated by phosphorylation of ERK in the TEY motif [14], [15] and may be altered through phosphorylation on other putative residues [16]–[18]. ERK nuclear targeting may also be altered through stimulus-dependent modification of the ERK binding partner repertoire. Accordingly, a recent proteomic study showed that the cast of ERK associated proteins is highly stimulus-dependent and dynamic [19]. ERK employs a modular docking domain system to ensure specificity of binding to partner proteins [20]. The best characterised of these are the negatively charged common docking (CD) motif opposite the catalytic site, which associates with positively charged D (docking)-domains in partner proteins [21], and the DEF-binding pocket (DBP) adjacent to the catalytic site, which binds to hydrophobic DEF (docking site for ERK, F/Y-X-F/Y-P) domains in target proteins [20], [22]. Mutation of D319N and Y261A residues of ERK2 impairs association with D- or DEF-domain containing proteins, respectively, without affecting TEY phosphorylation by MEK [23], [24]. These mutations are therefore useful for examining how protein association via these motifs influences ERK signal output in intact systems.

We recently used high-throughput single cell imaging techniques to show that both ERK TEY phosphorylation and relocalization to the nucleus were graded in proportion to epidermal growth factor (EGF) or phorbol 12, 13 dibutyrate (PDBu) stimulus level [24]. This contrasts with predictions that digitization of signalling may occur at the level of ERK nuclear traffic [25]. Surprisingly, we also found that MEK-directed phosphorylation of ERK is necessary but not sufficient to cause the full nuclear accumulation response. We further showed that redistribution of ERK to the nucleus was not proportional to the level of TEY phosphorylation, but was instead dictated by the nature and concentration of stimulus. Thus, nuclear localisation of ERK can be uncoupled from TEY phosphorylation level in the presence of stimulus, revealing a TEY phosphorylation “unattributable” component of the nuclear localization response. We have further demonstrated that this arm of the response is specifically dependent upon D-domain mediated binding of partner proteins to ERK [24]. In the current study, our primary aim was to establish whether a G-protein coupled receptor (GPCR) could also elicit this additional component of the ERK translocation response. We focused on GnRHR for three reasons. First, GnRHR mediated ERK activation can occur through several different signalling intermediates [2], [3], but the contribution of these components to ERK nuclear targeting is unclear. Second, GnRH mediated ERK signalling is essential for fertility [4], demonstrating the physiological importance of this pathway. Third, type I mammalian GnRHR have no C-terminal tail [1], [26], a structure implicated in agonist-dependent phosphorylation, arrestin binding and arrestin-dependent ERK activation for many 7TM receptors [6], [27]. Consequently, they do not show agonist-induced phosphorylation [28], do not bind arrestins [29]–[31] and do not rapidly desensitize or internalize [28], [30], [32]–[34]. They also do not show the switch from G-protein-mediated to arrestin-mediated ERK activation seen for other 7TM receptors [31] and therefore provide a relatively simple model for exploring 7TM receptor-mediated ERK activation mechanisms without the complication of rapid homologous desensitization or arrestin-mediated signaling.

Here, we show that GnRH induces redistribution of ERK from the cytoplasm to the nucleus and that this effect can be uncoupled from GnRH-mediated TEY phosphorylation of ERK. We also explore the mechanisms underlying this TEY phosphorylation unattributable component of the ERK translocation response.

## Methods

### Engineering of Plasmids and Viruses

Viral shuttle vectors were constructed initially by subcloning ERK2-GFP (a gift from Prof. Louis Luttrell, Medical University of South Carolina, Charleston, USA) and mouse GnRHR (a kind gift from Prof. Robert Millar, University of Edinburgh, UK) cDNAs into corresponding digests of pacAd5CMV K-N pA (donated by Prof. Beverly Davidson, University of Iowa, Iowa City, USA). K52R, T183/Y185A, Y261A and D319N mutations were introduced using a QuikChange PCR-based mutagenesis kit (Stratagene, Amsterdam, NL) and the following primers: 5′-CAA AGT TCG AGT TGC TAT CAG GAA AAT CAG TCC TTT TGA GC-3′, 5′-TGA TCA TAC AGG GTT CTT GGC AGA GGC TGT AGC CAC GCG TTG GTA C-3′, 5′-AAT TTA AAA GCT AGA AAC GCT TTG CTT TCT CTC CCG CAC-3′ and 5′-GCA GTA TTA TGA CCC AAG TAA TGA GCC CAT TGC TGA AGC-3′ along with antisense primers according to manufacturer’s recommendations and using the pacAd5CMV ERK2-GFP vector as the template. Viruses were generated from shuttle vectors as described [24], [35]. Verification of recombination was performed by restriction digest and sequence analysis, and Ad vectors were grown to high titre and purified according to standard protocols.

### Cell Culture and Transfection

HeLa cells were cultured in 10% FCS-supplemented Dulbecco’s modified Eagle’s medium (DMEM) without sodium pyruvate. For 96-well plate experiments, cells were harvested by trypsinization and seeded at 3−5×10^3^ cells/well. Where necessary, cells were transfected using RNAiMAX reagent (Invitrogen, Paisley, UK) and the manufacturer’s reverse transfection protocol. Cells were transfected with 2 siRNA duplexes (Qiagen, Crawley, UK) each for ERK1: 5′-CGU CUA AUA UAU AAA UAU AdTdT-3′, 5′-UAU AUU UAU AUA UUA GAC GdGdG-3′ and 5′-CCC UGA CCC GUC UAA UAU AdTdT-3′, 5′-UAU AUU AGA CGG GUC AGG GdAdG-3′ and for ERK2: 5′-CAC UUG UCA AGA AGC GUU AdTdT-3′, 5′-UAA CGC UUC UUG ACA AGU GdTdT-3′ and 5′-CAU GGU AGU CAC UAA CAU AdTdT-3′, 5′-UAU GUU AGU GAC UAC CAU GdAdT-3′ which have been validated in recent publications [23], [24], [36], [37]. A mixture of all 4 ERK duplexes or control siRNA against GFP (Ambion, Warrington, UK) was used in experiments at 2.5 nM total concentrations. Sixteen hours after siRNA transfection, cells were transduced with 0 or 1.5×10^6^ plaque-forming units (pfu)/ml Ad wild-type (WT), K52R, T183/Y185A, Y261A or D319N ERK2-GFP vector in DMEM with 2% FCS. The Ad-containing medium was removed after 4–6 hours and replaced with fresh DMEM supplemented with 0.1% FCS. The cells were then maintained for 16–24 h in culture prior to stimulation with GnRH (Sigma), EGF (Calbiochem, San Diego, CA, USA) or PDBu (phorbol 12, 13-dibutyrate, Sigma). In some experiments, cells were treated with 10 µM PD184352 (Enzo Life Sciences, Exeter, UK), 1 µM PD0325901 (Stratech Scientific, Newmarket, UK), 200 nM Ro31-8425 (Roche, Welwyn Garden City, UK), 100 nM AG1478 (Roche) or 30 µM cycloheximide (Sigma) for 10 min prior to stimulation. Expression levels of GFP-tagged fusions were compared using immunoblotting as well as comparison of mean cell fluorescence in microscopy assays.

### Immunoblotting

HeLa cells were plated in 6-well plates at 2.5×10^5^ cells/well. Where required, cells were transfected with 2.5 nM siRNAs targeting ERK1 and ERK2 prior to Ad transduction as above. Following treatment noted in figure legends, cells were lysed as described, prior to immunoblotting. Total and dual phosphorylated ERK (ppERK) were detected using rabbit anti-ERK monoclonal (clone 137F5, 1∶1000; Cell Signaling Technology, Hitchin, UK) and mouse anti-ppERK monoclonal antibody (clone MAPK-YT, 1∶2000, Sigma), respectively. Ser217/221 phosphorylated MEK1/2 were detected using a rabbit anti-ppMEK1/2 monoclonal antibody (clone 41G9, 1∶1000, Cell Signaling Technology). Antibodies were visualized using horseradish peroxidase linked secondary antibody and enhanced chemiluminescence kit (GE Healthcare, Amersham, UK).

### High-content Image Acquisition and Analysis

Cells were plated (and transfected with siRNA, and Ad vectors as necessary) as described above on Costar plain black-wall 96-well plates (Corning, Arlington, UK) prior to treatment as noted in figure legends. Cells were fixed in 4% paraformaldehyde/PBS and permeabilized in −20°C methanol. After blocking in 5% normal goat serum/PBS, cells were probed with mouse anti-ppERK monoclonal antibody (clone MAPK-YT, 1∶200, Sigma) and rabbit anti-ERK monoclonal (clone 137F5, 1∶100, Cell Signaling Technology) in PBS. Alexa 488-conjugated goat anti-mouse and Alexa 546-conjugated goat anti-rabbit secondary antibodies (1∶200, Invitrogen) and DAPI/PBS (600 nM) were used to visualize ppERK and ERK antibodies and to stain nuclei, respectively in imaging of endogenous kinases. For imaging cells transduced with Ad ERK2-GFP, cells were counterstained with rabbit anti-ERK monoclonal (1∶200) and Alexa 546-conjugated goat anti-rabbit secondary (1∶200) for assessing expression levels and setting filter levels. For imaging ppERK2-GFP in cells expressing ERK2-GFP, cells were counterstained with mouse anti ppERK monoclonal (1∶200) and Alexa 546-conjugated goat anti mouse secondary (1∶200) as above. Image acquisition in each well was performed on an IN Cell Analyzer 1000 (GE Healthcare) microscope, using a ×10 objective and 360 nm (DAPI), 475 nm (Alexa 488 and GFP) and 535 nm (Alexa 546) excitation filters, and monitored through 460 nm, 535 nm and 620 nm emission filters, respectively, with a 61002 trichroic mirror (GE Healthcare). Analysis of endogenous ERK and ppERK was performed using the Multi-target Analysis algorithm and “region growing” cell identification module in the IN Cell Analyzer Workstation (GE Healthcare) using DAPI and ERK stained images to define nuclear and cytoplasmic regions, respectively, which were used as a mask for detection of changes in ppERK staining. ERK2-GFP and ERK or ppERK stained cells were similarly analysed using the Multi-target Analysis algorithm, but using the “multiscale top hat” cell identification module (IN Cell Analyzer Workstation, GE Healthcare). In both ppERK2-GFP and GFP readouts, cells expressing sub- or super-physiological levels of ERK2-GFP were excluded from analysis (based on comparisons of frequency histograms of ERK staining intensity in cells transfected with control siRNA, ERK siRNAs and with ERK siRNAs plus Ad ERK2-GFP). Mitotic and apoptotic nuclei were excluded from analysis using a “decision tree” filter based on multiple readouts of intensity and shape defined in nuclear and cellular regions. Imaging data are reported as ppERK intensity in arbitrary fluorescence units (AFU per cell) or as a ratio of nuclear to cytoplasmic stain intensity (N:C ratio). Sorting of cells into linked subpopulations according to ppERK staining intensity was performed using a macro in Microsoft Excel.

## Results

### Using Immunoblotting and High-content Microscopy to Measure GnRH Effects on ERK

**Figure 1:**
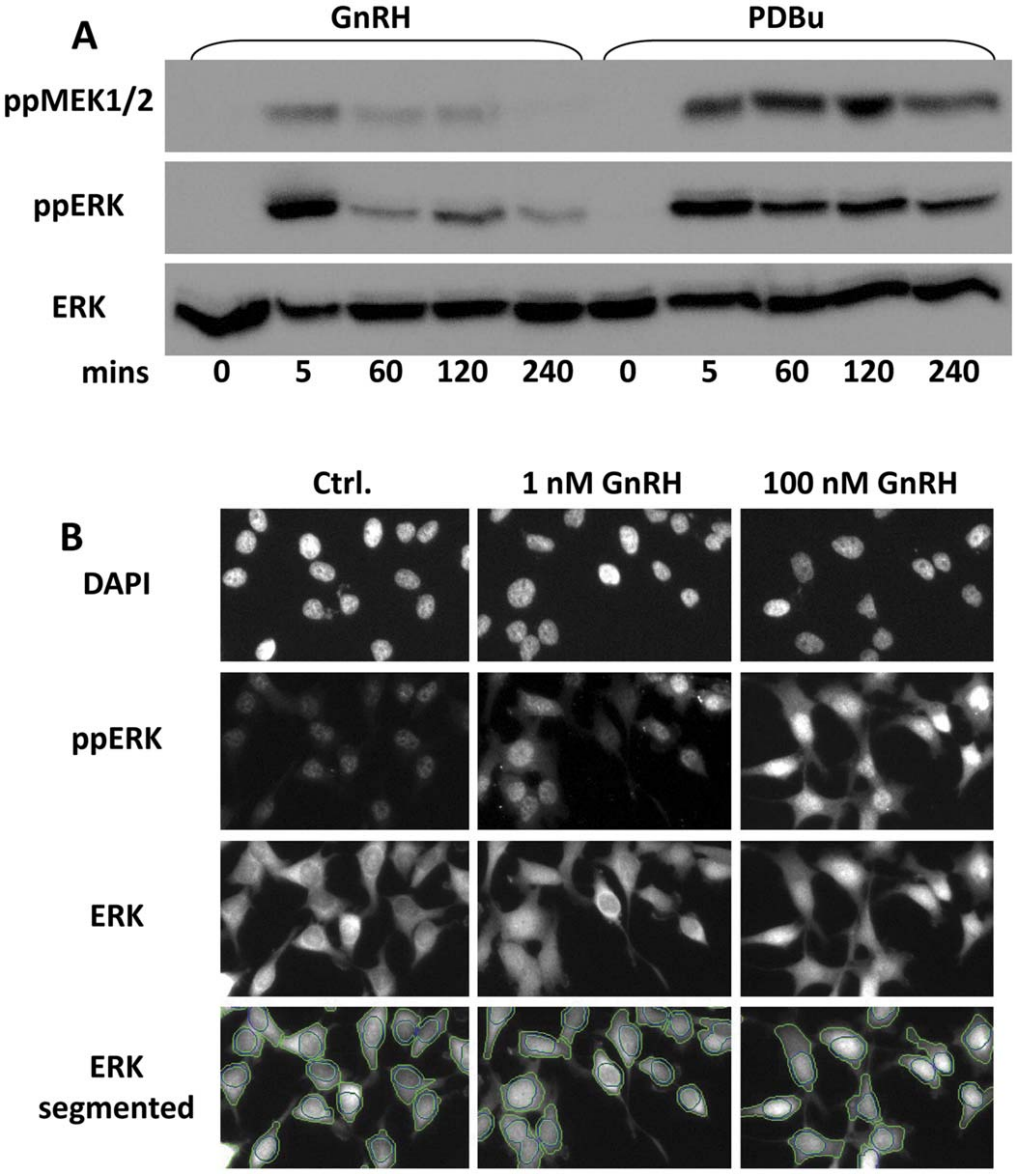
GnRH-stimulated MEK-ERK measured by western blotting or high content imaging. (A) HeLa cells were seeded in 12 well plates, transduced with Ad mGnRHR and kept in reduced (0.1%) serum for 16 hours prior to stimulation for varied periods with 100 nM GnRH or 1 µM PDBu. Whole cell protein extracts were then immunoblotted for phospho Ser217/221 MEK1/2 (ppMEK1/2), phospho Thr183/Tyr185 ERK (ppERK) and for total ERK (ERK1 and/or 2) as indicated. (B) HeLa cells were seeded in 96-well imaging plates, transduced with Ad mGnRHR and kept in reduced (0.1%) serum for 16 hours prior to stimulation for 5 min with 1 or 100 nM GnRH as indicated. Control cells (Ctrl.) were treated with medium alone. The cells were then fixed and stained for endogenous ppERK, ERK and DAPI before image acquisition and analysis (as described in the Methods). The figure shows representative images and the lower images show “segmented” ERK staining, with lines indicating the perimeters of the nuclei and cells obtained using automated image analysis algorithms, as described in the Methods. Each of the image panels corresponds to a width of approximately 250 µm and represents approximately 1/20^th^ of the area captured in each field of view.

**Figure 2:**
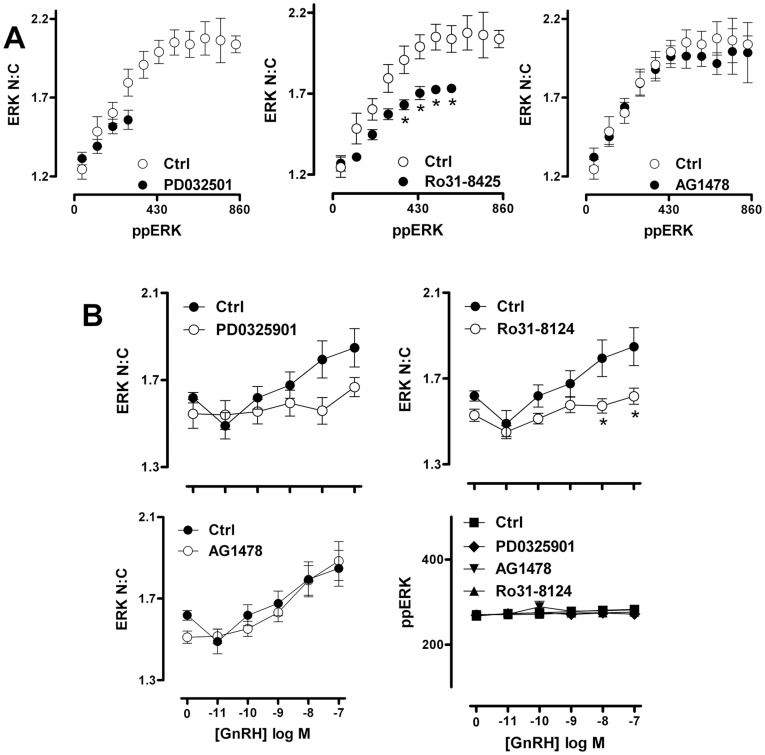
GnRH-stimulated MEK-ERK regulation in cell populations. HeLa cells were cultured, transduced, stimulated with 0, 1 nM or 100 nM GnRH and then fixed and stained as described under Fig. 1. Images of endogenous ppERK, ERK and DAPI stains were analysed (as described in Methods), using 9 images for each fluorophore and in each well, with cells in duplicate wells for each experiment. Graphs represent population average values for ppERK intensity (in arbitrary fluorescence units (AFU), left panels) and ERK N:C ratio (derived from AFU measures of total ERK stain intensity in the nuclear and cytoplasmic compartments) derived from 8 (A) and 14 (B) separate experiments ± SEM. Two way ANOVAs of data in panel A revealed GnRH as a significant source of variation (P<0.01) and post-hoc Bonferroni tests revealed significant differences between control and GnRH-treated cells as indicated (*P<0.05, **P<0.01). One way ANOVA of data in panel B revealed GnRH as a significant source of variation (P<0.01) and post-hoc Bonferroni tests revealed significant differences to control as indicated (*P<0.05, **P<0.01).

Most established culture models of gonadotrope cells are derived by SV40 T antigen-mediated immortalization [38]. As this is known to affect phosphatases that control the duration of ERK responses [39], we opted to study ERK signalling in a model HeLa cell system, using Ad to express murine GnRHR within physiological ranges of expression. We first determined the time course of ERK activation by immunoblotting for TEY phosphorylated ERK1 and/or ERK2 (ppERK) and total ERK in HeLa cells transduced with mGnRHR and stimulated for 0 or 5–240 min with 100 nM GnRH or 1 µM PDBu (Fig. 1A). This revealed rapid and transient activation by GnRH (maximum response at 5 min followed by rapid reduction to a near basal value at 60 min). In contrast, PDBu stimulation caused peak phosphorylation at 5 min followed by a lower, sustained plateau of ppERK levels (comparable ppERK levels at 60–240 min), confirming previous work in this model [24], [36], [37]. We also monitored dual phosphorylated MEK1/2 levels (as a readout for MEK activation) and this also revealed a rapid and transient effect of GnRH (maximal at 5 min, near basal from 60 min) and a more sustained effect of PDBu (comparable levels at 5–240 min) suggesting that the GnRH response is transient because the input from MEK is transient (and that phosphatase activity is consistently high). In order to increase experimental throughput and obtain information on compartmentalization we performed similar experiments using fluorescence microscopy. Total ERK and ppERK were stained simultaneously and quantified using a high-content image platform for automated image acquisition and analysis (Fig. 1B shows representative images and image segmentation). Again GnRH (100 nM) caused a rapid and transient increase in whole cell ppERK levels, increasing to a maximum at 5 min and returning to a near basal level by 60 min. The effect was also dose-dependent as a smaller response (with similar kinetics) was seen in cells stimulated with 1 nM GnRH (Fig. 2A, left panel). These responses were mirrored by transient and dose-dependent redistribution of ERK from the cytoplasm to the nucleus, as measured by ERK nuclear:cytoplasmic (N:C) ratio (Fig. 2A, right panel). We also constructed dose-response curves for cells stimulated for 5 min with 0 or 10^−11^–10^−7 ^M GnRH, which revealed dose-dependent activation (ppERK) and nuclear accumulation (N:C ERK) with comparable EC_50_ values of approximately 1 nM (Fig. 2B).

### Cell Sub-population Analysis Reveals Uncoupling of ERK Phosphorylation from Nuclear Localization

**Figure 3:**
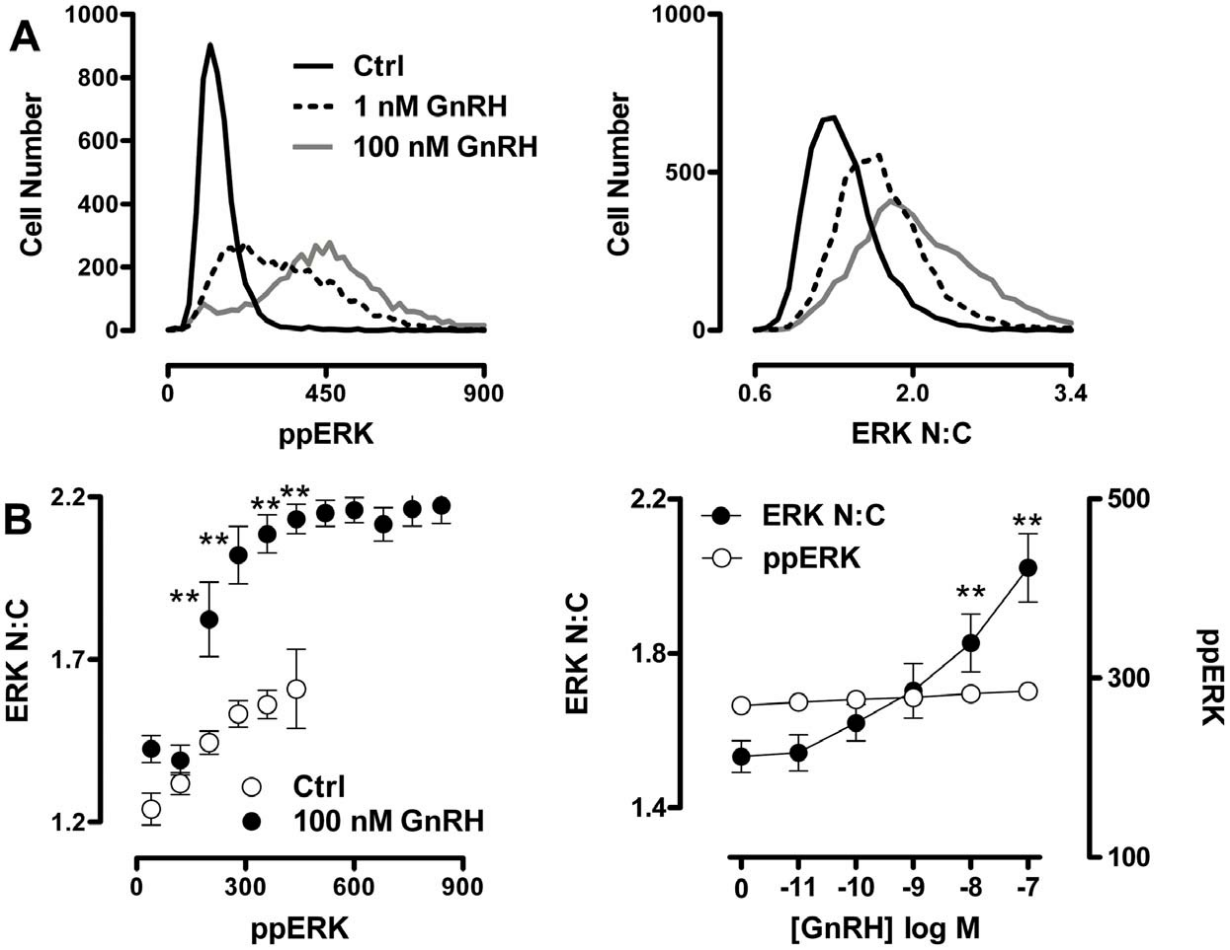
GnRH-mediated uncoupling of ERK phosphorylation from nuclear localization. Cells were treated, imaged and analysed as described for Fig. 2, except that the stimulation was for 5 min with varied concentrations of GnRH (as indicated). (A) Frequency histograms of individual cells (pooled from 2 independent experiments) were plotted according to ppERK stain intensity in AFU (left panel) and ERK N:C ratio (right panel) using the same cell population for both graphs. (B) The left panel shows direct comparison of ppERK levels to ERK N:C in Ctrl and 100 nM GnRH-stimulated samples. Individual cells were sorted into bins of ppERK staining intensity (80 AFU per bin, using a minimum bin size of 50 cells per experiment). The average ERK N:C ratio within each defined bin of ppERK staining intensity was calculated and is shown plotted against average ppERK stain intensity. Note that this plot effectively obscures the effect of the stimulus on ERK phosphorylation because the major effect of PDBu is to increase the number of cells in the higher ppERK bins (as shown in A). In doing so it reveals the TEY phosphorylation unattributable effect of PDBu: that is, the increase in ERK N:C under conditions matched for indistinguishable ppERK levels. The right hand panel illustrates the concentration-dependence of this effect with cells binned to ensure comparable levels of ppERK (240–320 AFU), plotting GnRH concentration against ERK N:C ratio (left y-axis)) and ppERK stain intensity (AFU, right y-axis). Data are shown from 13 separate experiments (mean ± SEM). Two way ANOVA of data in the lower left panel revealed both ppERK bin and GnRH concentration as significant sources of variation (P<0.01) and post-hoc Bonferroni tests revealed significant differences between control and GnRH-treated cells in the 200, 280, 360 and 440 AFU bins (**P<0.01). Since this analysis does not permit unpaired data, only data from the first six ppERK bins were included. One way ANOVA of the ppERK data in the lower right panel revealed GnRH concentration as a significant source of variation (P<0.01) and post-hoc Bonferroni tests revealed a significant difference between control and 10 or 100 nM GnRH-treated cells (**P<0.01).

**Figure 4:**
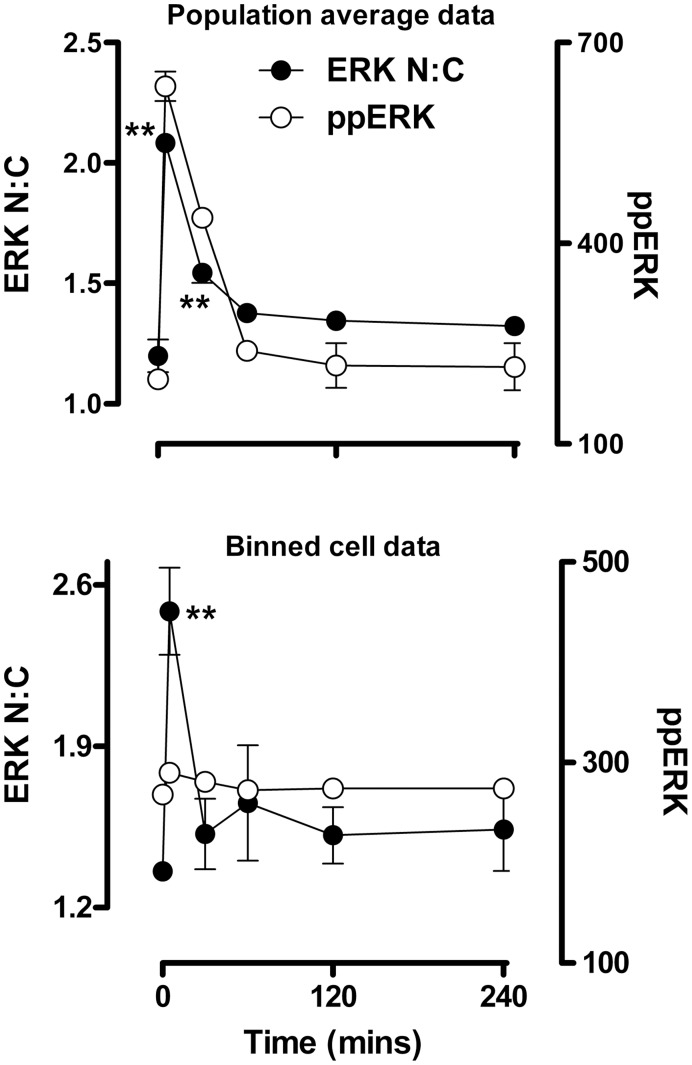
Time-dependent changes in GnRH-induced ERK localization occur at matched levels of TEY phosphorylation. Cells were treated, imaged and analysed as described for Fig. 2, except that the GnRH concentration was 100 nM. The top graph shows population average values for ppERK intensity in AFU (right y-axis) and ERK N:C ratio (left y-axis). The lower graph shows the same time-course, but comparing ERK N:C ratio (left y-axis) in cells within a comparable range (240–320 AFU) of ppERK staining intensity (right y-axis). Data are shown from 3 separate experiments (mean ± SEM). One way ANOVAs and post-hoc Bonferroni tests revealed significant effects of GnRH on ERK N:C (**P<0.01 compared to t = 0) at 5 and 15 min for the population average data (upper panel) and at 5 min for the binned data (lower panel).

Previous studies, including work from our own laboratory, has shown that population average data derived from thousands of imaged or lysed cells (such as that shown in Fig. 2) can mask the true relationship between ERK phosphorylation and nuclear localization [24], [36], [40]. In order to examine how GnRH stimulus changes ERK signalling dynamics, we interrogated data recorded from single cells in high content microscopy experiments. When HeLa cells expressing GnRHRs were stimulated for 5 min with 0 , 1 nM or 100 nM GnRH, frequency distribution plots of single cell data revealed that the hormone causes dose-dependent increases in both the proportion of cells responding to GnRH and the mean response in those cells (Fig. 3A). This is consistent with the mixed digital/graded ERK responses previously seen in GnRHR expressing HeLa cells and in LβT2 cells [41]. A further intriguing feature of these data is that whole cell ppERK values were broadly spread (99% of 100 nM GnRH-stimulated cells within a 900-fold range of ppERK levels) (Fig. 3A, left panel) as compared to the much tighter N:C ERK range (99% of 100 nM GnRH-stimulated cells within 6-fold N:C ERK range) (Fig. 3A, right panel). This suggests uncoupling of the responses, with relatively constant nuclear localization over a broad range of ppERK levels. To test for this, we sorted the cells into bins according to ppERK level (each spanning 80 AFU) and for each bin, plotted the mean ppERK value against the mean ERK N:C ratio. In control cells this revealed a positive correlation between the ppERK and N:C ERK measures (Fig. 3B, left panel). In contrast, when cells were treated for 5 min with 100 nM GnRH a different relationship was observed with the N:C ERK ratio increasing to a maximum of approximately 2.2. Importantly, ERK N:C levels were greater than that of control cells in almost all comparable ppERK bins (Fig. 3B, left panel). A similar uncoupling of responses was seen in cells stimulated for 5 min with 1 nM GnRH (not shown), and when a single bin size (ppERK 240 to 320 AFU) was used to monitor whole cell ppERK levels in cells stimulated for 5 min with varied doses of GnRH (Fig. 3B, right panel). Using the same approach to establish the time-course of GnRH effects, we observed the expected transient increase in whole cell ppERK and N:C ERK when the entire cell population was measured (Fig. 4A). However, we also saw a transient increase in ERK N:C using a single bin of cells in which whole cell ppERK levels were 240 to 320 AFU (Fig. 4B). Uncoupling of ERK phosphorylation from translocation has been observed in several models and has been attributed to neosynthesis of nuclear dual-specificity phosphatases (DUSPs) that can anchor and dephosphorylate ERK within the nucleus [36], [37], [42], [43]. We have previously shown that GnRHR activation in this model increases transcription of nuclear-inducible DUSP1, 2, 4 and 5 [44], [45] but GnRH effects on the corresponding mRNA levels are not seen in early (<30 min) phases of signalling. Further, we saw no change in the relationship between ERK phosphorylation and nuclear localization after 5 min stimulus in the presence of protein synthesis inhibitors (not shown), suggesting that DUSP neosynthesis is unlikely to be relevant for the rapid uncoupling seen here with GnRH.

**Figure 5:**
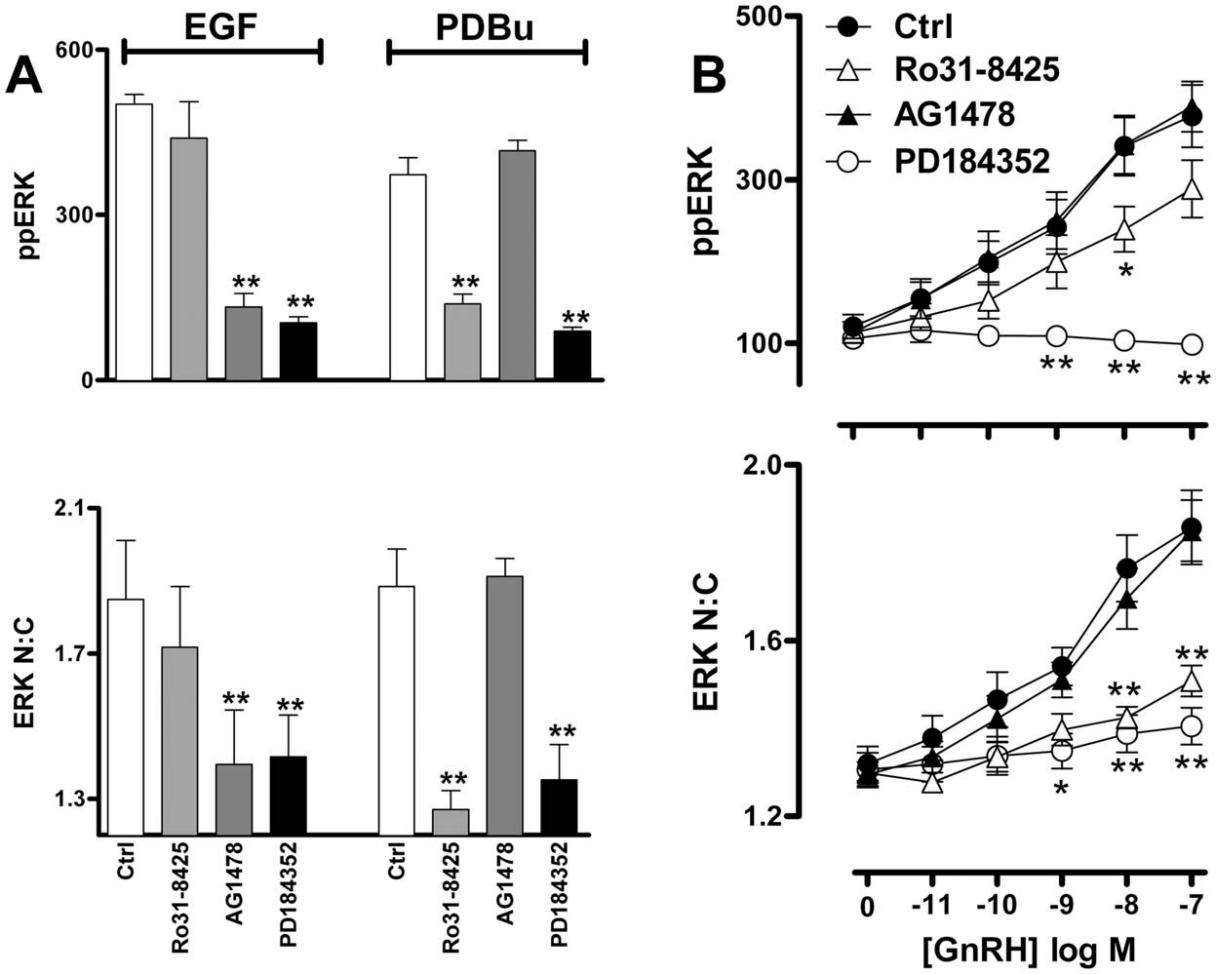
GnRH-induced ERK phosphorylation and nuclear localization is partially dependent on PKC activation. Cells were treated, imaged and analysed as described for fig. 2, except that the stimulation was for 10 min with 1 µM PDBu or 10 nM EGF in cells that had been pre-treated for 10 min with DMSO vehicle (Ctrl), 200 nM Ro31-8425 PKC inhibitor, 100 nM AG1478 EGFR inhibitor or 10 µM PD184352 MEK inhibitor (panel A), or the cells were stimulated for 5 min with the indicated concentrations of GnRH in the presence of the same inhibitors (panel B). Panel A show population average values for ppERK in AFU (top panel) and ERK N:C (bottom panel) after treatment with inhibitors and agonists as indicated. Data are shown are mean ± SEM (n = 3), ** = p<0.01, comparing Ctrl and inhibitor co-incubations for each readout, according to one-way ANOVA and Dunnet’s post-hoc test. Panel B shows concentration-response curves for the effect of GnRH on population averaged ppERK intensity (in AFU) and ERK N:C responses. Data are shown means ± SEM (n = 6) and two way ANOVAs revealed GnRH, Ro31-8425 and PD184352 as significant sources of variation (P<0.01) for both measures (ppERK and ERK N:C), whereas AG1478 was not a significant variable for either (P>0.05). *P<0.05, **P<0.01 comparing with and without inhibitor at matched GnRH concentration.

**Figure 6:**
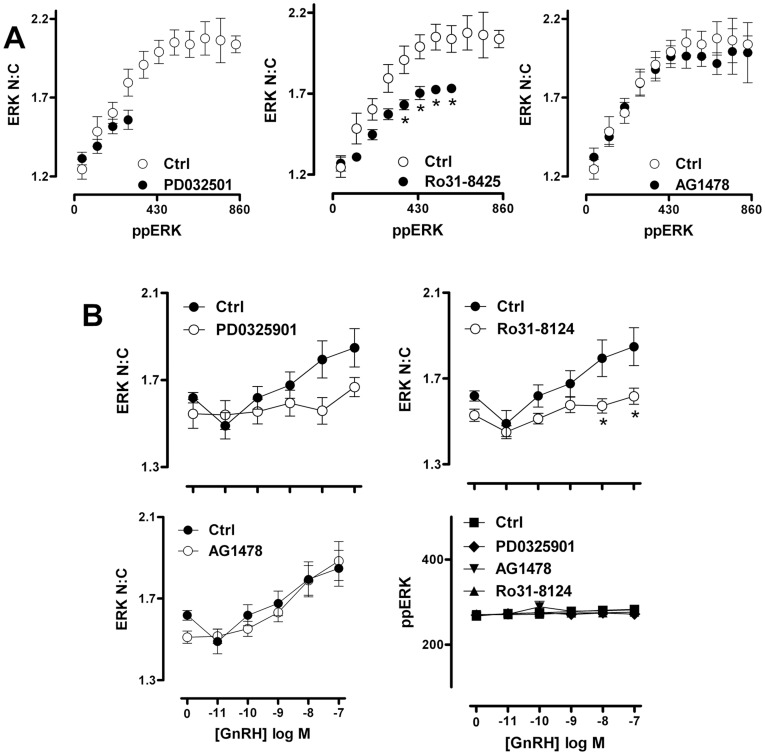
PKC inhibition reduces GnRH-induced nuclear localization of ERK even at comparable levels of ERK TEY phosphorylation. Cells were treated, imaged and analysed as described under fig. 5, except that stimulation was for 5 min with 100 nM GnRH (panel A) or with GnRH at varied concentration (panel B). (A) The individual imaged cells were sorted into bins of ppERK staining intensity (80 AFU per bin, using a minimum bin size of 50 cells per experiment). The average ERK N:C ratio within each defined bin of ppERK staining intensity was then calculated and is shown plotted against average ppERK stain intensity (in AFU). The data are shown are means ± SEM (n = 6) and two way ANOVAs revealed ppERK bin, PD032501 and Ro31-8425 as significant sources of variation (P<0.01), whereas AG1478 was not (P>0.05). Post-hoc Bonferroni tests revealed significant differences between ERK N:C values in control and Ro31-8425 treated cells in 4 of the ppERK bins (*P<0.05). Only paired data (i.e. bins where data are available with and without inhibitor) were used in this analysis. (B) To reveal whether inhibitor effects in cells at matched ppERK levels were dependent on GnRH dose, we used data from single cells to comparing ERK N:C ratio (left y-axis) in cells within a comparable range (240–320 AFU) of ppERK staining intensity (in AFU, right y-axis) for each GnRH concentration tested. Data are means ± SEM (n = 6). Two way ANOVAs of the ERK N:C data revealed GnRH, Ro31-8425 and PD0325901 as significant sources of variation (P<0.01) whereas AG1478 was not (P>0.05). Post-hoc Bonferroni tests revealed significant differences between ERK N:C values in control and Ro31-8425 treated cells at 10 and 100 nM GnRH (*P<0.05).

Together, the data above show that mechanisms other than phosphorylation-dependent release from MEK or agonist-induced up-regulation of nuclear DUSPs are needed to achieve the full ERK nuclear localization response elicited by activation of a GPCR. Similar conclusions were reached in our previous study exploring ERK translocation in response to PKC or EGF receptor activation [24]. Since activation of ERK by GnRH is mediated by PKC and/or EGF receptor transactivation in other models, we explored their possible involvement in the TEY phosphorylation unattributable translocation response. Using whole cell population measures we found that PDBu and EGF induced an expected increase in ppERK and N:C ERK (Fig. 5A). The MEK inhibitor PD184352 inhibited both responses to EGF and PDBu whereas inhibition of EGF receptor activation with AG1478 only inhibited the responses to EGF, while inhibition of PKC with Ro31-8425 exclusively caused pronounced inhibition of the PDBu effect (Fig. 5A). This confirms the specificity and efficacy of these inhibitors against target pathways in this context. In order to explore the contribution of EGF receptor and PKC signalling to GnRH-mediated ERK activation, we employed similar inhibitor protocols to generate GnRH dose-response curves. These revealed (Fig. 5B) that GnRH effects on whole cell ppERK and N:C ERK were abrogated by PD184352 (and a more potent PD0325901 MEK inhibitor) but were only partially inhibited by Ro31-8425 (at GnRH doses of 10^−11^–10^−7^ M) and not influenced by AG1478 (at any GnRH dose). Interestingly, the Ro31-8425 inhibitor reduced ERK N:C responses to a greater degree than ppERK response in the same cells (compare Fig. 5B, top and bottom panels). We also examined frequency distribution curves for ppERK and N:C ERK in individual cells stimulated with 10^−9^ or 10^−7^ M GnRH under control conditions or in the presence of PD0325901, Ro31-8425 or AG1478. This revealed that whole cell ppERK values were broadly spread, as compared to the much tighter N:C ERK range (confirming the relationship shown in Fig. 3A) and that this distinction was retained in cells stimulated in the presence of AG1478 but was reduced in cells stimulated in the presence of Ro31-8425 or PD0325901 (not shown). To test this more directly, we calculated N:C ERK values for cells binned according to their ppERK values. This revealed the expected relationship in GnRH-stimulated cells, with N:C ppERK values showing a monotonic rise to a plateau of approximately 2.2 as ppERK values rise (from a bin centre of 40 to 860 AFU) (Fig. 6A, see also fig. 3B). This relationship was unaltered by AG1478 (Fig. 6A, right panel) but the plateau was markedly reduced (to approximately 1.7) in cells treated with GnRH in the presence of Ro31-8425 (Fig. 6A, middle panel). The MEK inhibitor PD0325901 greatly reduced ppERK levels so that bins could not be created for cells having high ppERK levels and the plateau could not be defined under these conditions (Fig. 6A, left panel). We also constructed full GnRH dose-response curves in the presence and absence of these inhibitors using a single ppERK bin (i.e. including only cells in which then ppERK was 240 to 320 AFU). As shown (Fig. 6B), GnRH induced the expected dose-dependent increase in ERK N:C (without any measurable increase in ppERK). This effect was not inhibited by AG1478 but was significantly reduced by the MEK inhibitor and the PKC inhibitor. Thus, PKC inhibition reduces ERK nuclear translocation even at comparable levels of TEY phosphorylation. This suggests that PKC activation causes nuclear localization of ERK by additional mechanisms to TEY phosphorylation. These data echo earlier work showing that GnRH-stimulated ERK activation is independent of EGF receptor transactivation and partially dependent on PKC activation in this model, and that the PKC-dependent pool of phosphorylated ERK constitutes the fraction able to translocate to the nucleus [31]. They are also consistent with reports showing that PKC mediated phosphorylation of the ERK cytoplasmic anchor, protein enriched in astrocytes-15 (PEA-15), is required for nuclear localization of ERK in LβT2 cells [46].

### GnRH-induced Uncoupling of ERK Phosphorylation from Translocation is Dependent on D-domains

**Figure 7:**
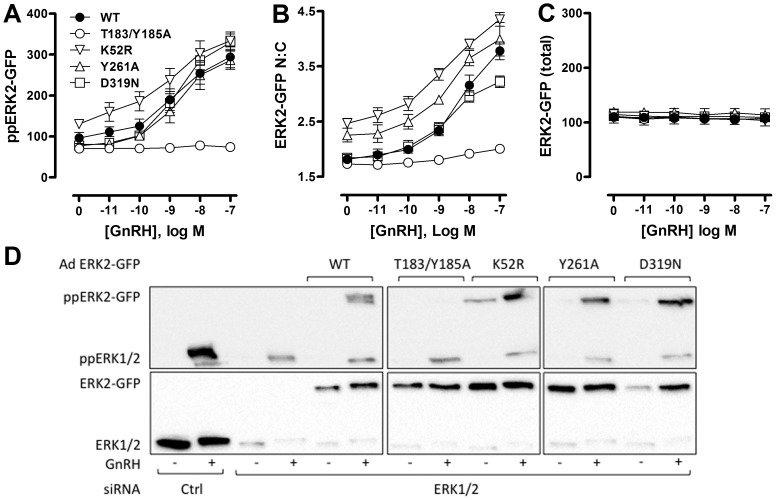
Phosphorylation, catalysis and docking domains control ERK targeting in response to GnRH. HeLa cells transfected with ERK siRNAs (to knock-down endogenous ERK) and were transduced with Ad to add-back wild-type (WT), K52R, T183/Y185A, Y261A or D319N-mutated ERK2-GFP. They were then stimulated with GnRH as indicated for 5 minutes before fixation, ppERK2-GFP staining, image acquisition and analysis as described in Methods, to assess whole-cell levels of TEY-phosphorylated ERK2-GFP (ppERK2-GFP in AFU, panel A), nucleo-cytoplasmic distribution of ERK2-GFP (ERK2-GFP N:C, panel B) and whole-cell levels of ERK2-GFP in the nucleus and cytosol (total ERK2-GFP, panel C). Data are population average means ± SEM (n = 7) Two way ANOVAs of data in panel A revealed GnRH as a significant source of variation, as were the T183/Y185A and K52R mutations (P<0.05) and post-hoc Bonferroni tests revealed significant differences between WT and T183/Y185A-ERK2 expressing cells at 1, 10 and 100 nM GnRH (P<0.05). Two way ANOVAs of the panel B data revealed GnRH and all 4 mutations as significant variables (P<0.01). Post-hoc Bonferroni tests revealed significant differences (P<0.05) between WT- and T183/Y185A-ERK2 expressing cells at 1, 10 and 100 nM GnRH, between WT- and K52R-ERK2 expressing cells at all GnRH concentrations, between WT- and Y261A-ERK2 expressing cells at 1 nM GnRH, and between WT- and D319N-ERK2 expressing cells at 100 nM GnRH. Similar analysis of the panel C data revealed that neither GnRH nor mutant expressed were significant sources of variation (P>0.05). For panel D, HeLa cells cultured in 12 well plates were transfected with ERK siRNAs (to knock-down endogenous ERK) or with control siRNAs (Ctrl) and were transduced with Ad to add-back wild-type (WT), K52R, T183/Y185A, Y261A or D319N-mutated ERK2-GFP. They were then stimulated without or with GnRH (−/+, as indicated) for 5 minutes before whole cell protein extraction, and western blotting for ppERK and for total ERK (ERK1 and/or 2) as indicated. The data shown are from an experiment that is representative of 3 such experiments.

To further explore relationships between GnRH-mediated changes in ERK phosphorylation and localization, we used siRNAs to reduce expression of endogenous ERK, and recombinant Ad to introduce GFP-tagged wild-type (WT) or mutated ERK2 reporters. We have previously shown that the siRNA treatment reduces endogenous ERK levels by >95%, and that this is paralleled by reduced stimulated ERK phosphorylation (as measured by cell imaging or by immunoblotting) and Egr-1 Luc activity (early growth response-1 promoter-luciferase reporter, used as a transcriptional readout for nuclear ERK activity), and that addition of Ad ERK2-GFP restores ERK expression, stimulated ERK phosphorylation and Egr-1 Luc responses to wild-type levels [24], [36], [37]. A similar approach was used to add-back mutated ERK2-GFP constructs (after siRNA knock-down) and immunoblotting revealed comparable expression levels for WT ERK2-GFP and for reporters containing K52R (catalytically inactive), T183/Y185A (non-TEY phosphorylatable), Y261A (deficient in binding DEF domains) and D319N (deficient in binding D-domains) variants of ERK2-GFP, as did comparison of whole cell levels of GFP fluorescence (Fig. 7C and D). None of the mutations inhibited MEK expression or PDBu-stimulated MEK phosphorylation [24].

Imaging assays (population averaged data) were then used to define effects of these mutations on responses to GnRH. As shown, 5 minutes stimulation with GnRH caused a dose-dependent increase in ERK2-GFP phosphorylation (whole cell ppERK2-GFP, Fig. 7A) and nuclear localization (ERK2-GFP N:C; Fig. 7B). Monitoring whole cell expression levels (N+C ERK2-GFP intensity) confirmed that differences in ERK2-GFP N:C reflected genuine changes in localization rather than reporting differences in expression of GFP-tagged ERK2 (Fig. 7C). Both ppERK2-GFP and localization responses were markedly reduced in cells expressing the T183/Y185A ERK2-GFP mutant, confirming the dependence of nuclear localization on TEY phosphorylation [24], [47]. GnRH also dose-dependently increased phosphorylation and nuclear localization of the K52R mutant, confirming that the phosphorylation and nuclear localization responses do not require catalytic activity [24], [47], although this mutation does prevent activation of the Egr-1-luciferase transcription reporter in this model. As in our previous study, we found that basal ppERK2-GFP levels and ERK2-GFP N:C ratio were increased with this mutant (Fig. 7A and B), which likely reflects the lack of negative feedback requiring ERK catalytic activity [48]–[51].

**Figure 8:**
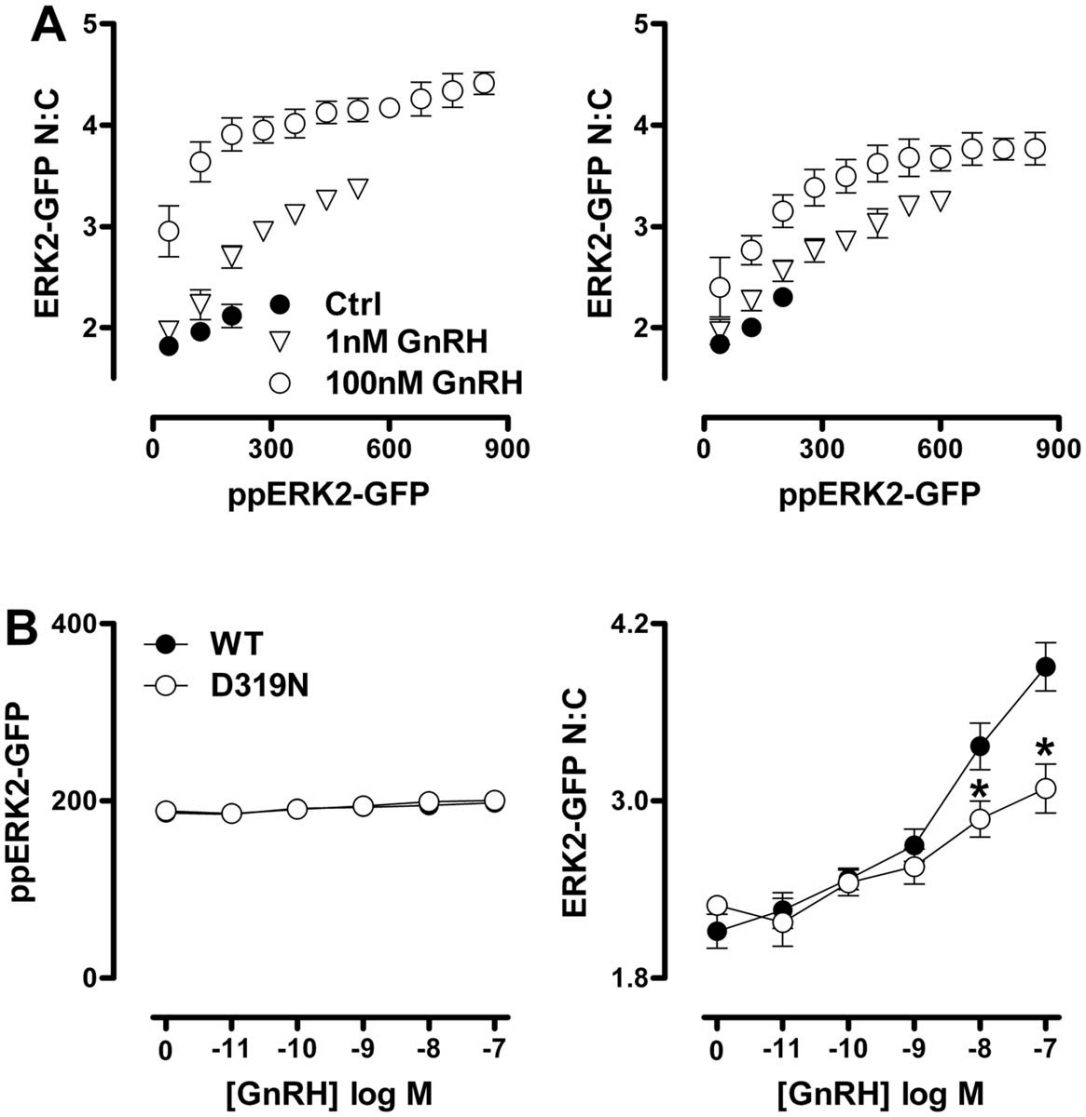
D319N mutation of ERK2-GFP inhibits GnRH-induced nuclear localization that is not attributable to increases in TEY phosphorylation. (A) HeLa cells transfected with ERK siRNAs were transduced with Ad wild-type (WT) or D319N-mutated ERK2-GFP as indicated and stimulated with vehicle (Ctrl), 1 nM or 100 nM GnRH for 5 minutes prior to staining, imaging and analysis as described in Methods. The plots show comparison of average ERK2-GFP N:C ratio in cell populations within defined bins of ppERK2-GFP staining intensity (80 AFU per bin, accepting a minimum of 50 cells per bin in each experiment). Data shown are mean ±SEM (n = 3). Two way ANOVAs revealed ppERK2-GFP as a significant source of variation for control cells and for cells receiving either GnRH concentration. The D319N mutation was a significant variable (P<0.05) only for cells receiving 100 nM GnRH and in this case, post-hoc Bonferroni tests revealed significant differences between WT- and D319N-ERK2 expressing cells in the lowest 2 ppERK2 bins (P<0.05). Only paired data (i.e. the three bins where data are available with and without inhibitor) were used in this analysis. (B) The graphs show full concentration-response curves of ERK2-GFP N:C ratio (right panel) for cells treated as in panel A, using only cells within a defined range (160–240 AFU) of ppERK2-GFP staining intensity present at all levels of stimulus (left panel). Data are mean ± SEM (n = 8). Two way ANOVA for the ERK2-GFP N:C data revealed both GnRH concentration and D319N mutation as significant variables (P<0.01) and post-hoc Bonferroni tests revealed significant differences between WT- and D319N-ERK2 expressing cells receiving 10 or 100 nM GnRH (*, P<0.05).

The Y261A mutation, which inhibits DEF domain-dependent binding, did not affect ERK2-GFP phosphorylation in response to GnRH but did increase N:C ERK2-GFP ratios in basal and stimulated cells (see also [24], [36] for similar data with PDBu treated cells). In contrast, inhibition of ERK2-GFP binding to D-domain partners (D319N mutation) did not measurably alter ppERK2-GFP under basal or GnRH-stimulated conditions, but it did have a marginal inhibitory effect on nuclear localization at high concentrations of GnRH. These data are consistent with earlier experiments showing that the D319N mutation modulates responses to sustained stimulation with PDBu, but has only a marginal effect on transient responses to GnRH [45]. We next asked whether the uncoupling of ERK phosphorylation from nuclear localization seen with endogenous ERK would also be seen with the knock-down/add-back model. To do so, endogenous ERK was knocked down with siRNA, recombinant adenovirus was used to add back ERK2-GFP and cells were incubated for 5 min with 0, 10 nM or 1 µM GnRH before imaging for ppERK2-GFP and ERK2-GFP. Binning the individual cells according to ppERK2-GFP levels revealed a positive correlation between ppERK2-GFP and N:C ERK2-GFP in control cells. Moreover, a marked uncoupling of the responses was seen when cells were stimulated with GnRH. Indeed, ERK2-GFP N:C values were increased (as compared to control) in all cell subsets with matched ppERK2-GFP level. These data are very similar to those seen with endogenous ERK (compare left panels of Figs. 3B and 8A).

The previous data show that the GFP tag does not interfere with the component of the nuclear localization response to GnRH that cannot be attributed to TEY phosphorylation, and this enables exploration of mechanisms with ERK2-GFP mutants. Accordingly, we compared the effect of the ERK2-GFP mutants on stimulus-dependent localization within matched ppERK2-GFP levels. This analysis clearly cannot be performed with the T183/Y185A mutant (which lacks the TEY phospho-acceptor sites). Very similar data showing clear uncoupling of nuclear localization from phosphorylation were obtained from cells expressing WT, K52R and Y261A ERK2-GFP and stimulated for 5 min with 1 or 100 nM GnRH (not shown). In contrast, the level of uncoupling of phosphorylation from nuclear localization seen in cells expressing D319N ERK2-GFP was far less pronounced than with the wild-type ERK2-GFP (compare left and right panels in Fig. 8A). This distinction was also evident in dose-response studies where data are plotted for the sub-set of cells within a single narrow ppERK2-GFP bin (Fig. 8B). This revealed a clear dose-dependent increase in N:C ERK2-GFP under conditions where there was no measurable GnRH-stimulated increase in ppERK2-GFP and the nuclear localization response seen with the binned data was significantly reduced in cells expressing D319N ERK2-GFP (Fig. 8B). Consistent with the population averaged data (Fig. 7) we also found that the K52R and Y261A mutants increased ERK2-GFP N:C ratios in the absence of stimulus and this was additive with the GnRH effects (not shown). These data demonstrate that the additional component of the GnRH-mediated ERK nuclear localization response that cannot be attributed to TEY phosphorylation, does not require catalysis or DEF domain-dependent binding, but is largely prevented by the D319N mutation and therefore requires association with D-domain containing partner proteins.

**Figure 9:**
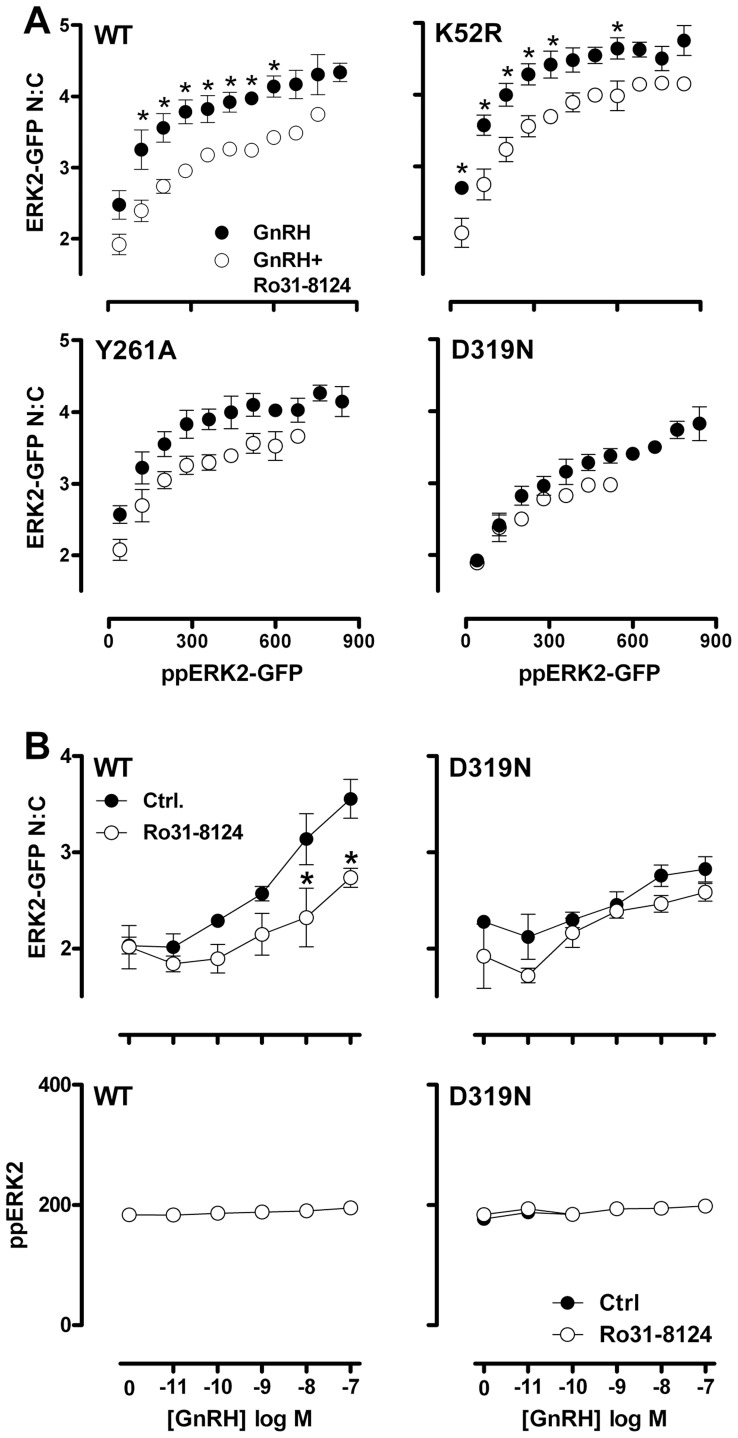
Effects of D319N mutation and PKC inhibition on TEY phosphorylation unattributable nuclear localization of ERK2-GFP are not additive. HeLa cells transfected with ERK siRNAs were transduced with Ad wild-type (WT) or D319N-mutated ERK2-GFP as indicated and incubated with vehicle (filled dots) or 200 nM Ro31-8425 PKC inhibitor (open dots) 10 minutes priors to stimulation with 100 nM GnRH for 5 minutes. Cells were then fixed, imaged and analysed as described in the Methods. The plots show comparison of average ERK2-GFP N:C ratio in cell populations within defined bins of ppERK2-GFP staining intensity (80 AFU per bin, accepting a minimum of 50 cells per bin in each experiment). Data are means ± SEM (n = 3). Two way ANOVAs revealed ppERK2-GFP bin as a significant source of variation for all conditions (P<0.01). Ro31-8425 was a significant variable for cells expressing WT-, K52R- or Y261A-ERK2 GFP (P<0.01), but not for cells expressing D319N ERK2 GFP (P>0.05). Post-hoc Bonferroni tests revealed significant effects of Ro31-8425 in cells expressing WT-ERK2-GFP or K52R ERK2-GFP (*, P>0.05). Only paired data (i.e. the bins where data are available with and without inhibitor) were used in this analysis. (B) The graphs show full GnRH concentration-response curves for cells transfected, transduced and treated with Ro31-8425 as described in (A) but stimulated for 5 minutes with varied concentrations of GnRH. We compared ERK2-GFP N:C ratio (right panel) in cells within a defined range (160–240 AFU) of ppERK2-GFP staining intensity present at all levels of stimulus. Data are mean ± SEM (n = 3). In two way ANOVAs of the ERK2-GFP N:C data, GnRH concentration and Ro31-8124 were significant variables. Post-hoc Bonferroni tests revealed significant effects of Ro31-8425 in cells expressing WT-ERK2-GFP and stimulated with 10 or 100 nM GnRH (P<0.05), but not under any other conditions (*, P>0.05).

We next explored the combined effects of ERK2-GFP mutation and inhibition of PKC using the Ro31-8425 compound. The Ro31-8425 inhibitor showed identical effects in WT ERK2-GFP expressing cells to the effects seen with endogenous ERK (Fig. 5B), in that non-stimulated cells were unaffected, and population average levels of ppERK2-GFP and ERK2-GFP N:C in GnRH-stimulated cells were substantially reduced (not shown). Similarly, when we compared binned data from WT ERK2-GFP expressing cells stimulated with 100 nM GnRH, we found that inhibition of PKC reduced the nuclear localization of ERK2-GFP in nearly all bins of ppERK2-GFP intensity (compare Fig. 9A, top left panel and Fig. 6A, middle panel). This was also apparent in full GnRH dose-response curves in the presence and absence of Ro31-8425 inhibitor comparing a single ppERK2-GFP bin (i.e. including only cells in which then ppERK2-GFP was 160 to 240 AFU; Fig. 9B, left panels and Fig. 6B), indicating that the GFP tag did not influence the effects of PKC inhibition on the ERK response. To test whether the GnRH-mediated, PKC-dependent uncoupling of ERK phosphorylation from nuclear localization was wholly or partially dependent on ERK catalysis or docking motifs, we compared nuclear localization at matched levels of TEY phosphorylation for WT, K52R, Y261A and D319N ERK2-GFP mutants in internally controlled experiments. PKC inhibition in GnRH-stimulated cells expressing K52R or Y261A mutated ERK2-GFP had similar effects to that seen in WT samples, in that the Ro31-8425 inhibitor reduced nuclear localization across a wide range of ppERK2-GFP levels (Fig. 9A) as well as when comparing full GnRH dose curves using a single ppERK2-GFP bin (not shown). This indicates that the PKC-dependent component of the GnRH-induced ERK2-GFP nuclear localization response is not dependent upon ERK2-GFP catalysis, or association with DEF-domain containing proteins. Strikingly however, we found that PKC inhibition had no additional effect on ERK2-GFP nuclear localization at matched levels of phosphorylation in D319N ERK2-GFP expressing cells (Fig. 9A, bottom right panel and Fig. 9B, right panels). This indicates that, while PKC inhibition reduces population averages of ERK nuclear localization in GnRH-stimulated cells expressing D319N ERK2-GFP, it does so simply by reducing the magnitude of TEY phosphorylation. This is in marked contrast to the effects of PKC inhibition on endogenous ERK and WT ERK2-GFP expressing cells, where PKC inhibition causes a substantial reduction in nuclear localization even when TEY phosphorylation levels are matched. Thus, PKC inhibition has a mechanistically distinct effect on ERK nuclear traffic in cells expressing WT and D319N-mutated ERK2-GFP.

**Figure 10:**
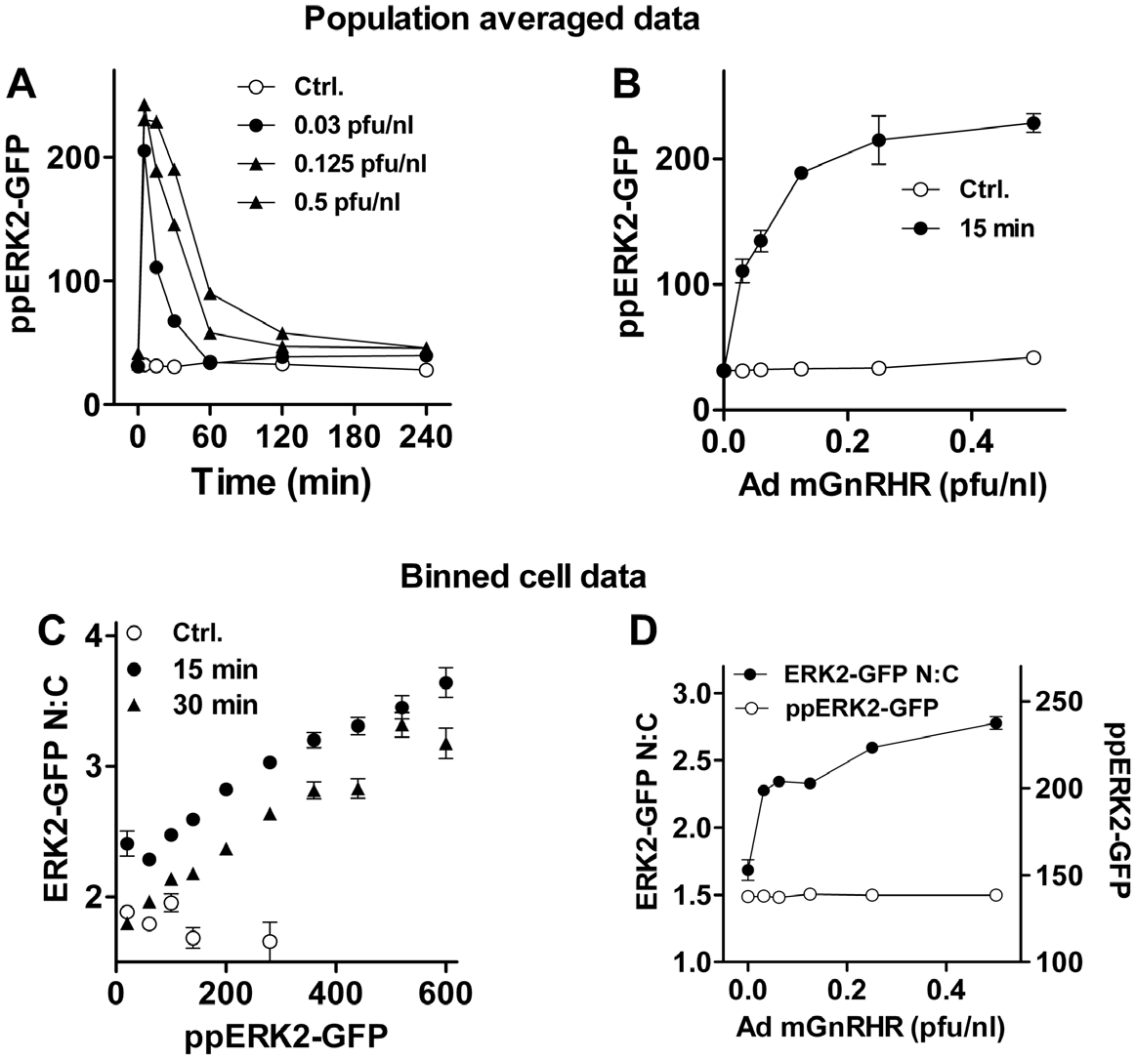
Relevance of GnRHR expression level for TEY phosphorylation unattributable nuclear localization of ERK2-GFP. For panels A and B, HeLa cells transfected with ERK siRNAs were transduced with Ad ERK2-GFP (WT) and with Ad mGnRHR at varied titre (0, 0.03, 0.06, 0.125, 0.25 or 0.5 pfu/nl) in order to vary GnRHR number. They were then stimulated for the indicated period before being fixed and stained for ppERK2-GFP and DAPI. Imaging and analysis of single cells was carried out as described in Methods. The plots show cell population averaged ppERK2-GFP levels for cells stimulated with 100 nM GnRH plotted against time (A), or for cells stimulated 15 min with 0 (Ctrl.) or 100 nM GnRH, plotted against Ad mGnRHR titre (B). These data are from a single experiment with duplicate wells, and are representative of those from 2 similar experiments. Panel C show ERK2-GFP N:C ratios calculated for data from the same experiment, binned according to ppERK2-GFP levels (80 AFU per bin, accepting a minimum of 50 cells per bin in each experiment). Data are shown for cells transduced with 0.25 pfu/nl Ad mGnRHR and stimulated for 0 (Ctrl.), 15 or 30 min with 100 nM GnRH. Panel D shows ppERK2-GFP values (AFU) and ERK2-GFP N:C ratios calculated for cells within a single ppERK2-GFP bin (120–160 AFU), plotted against Ad mGnRHR titre. ANOVAs of the ppERK2-GFP data in panels B and C both revealed Ad mGnRHR titre as a significant variable with significant elevation (P<0.01 compared to control without Ad mGnRHR) at all titres.

In the final series of experiments we considered the relevance of GnRHR number for the TEY phosphorylation unattributable ERK translocation response by using the ERK2-GFP knock-down/add back protocol in cells in which GnRHR was set by varying Ad mGnRHR titre. As shown, GnRH caused a transient increase in population averaged ppERK2-GFP levels, with increasing Ad mGnRHR titre increasing both the duration and the amplitude of the responses (Figs. 10A and 10B). From previous work with this model [44], [45] we estimate that the Ad mGnRHR titres used here (0, 0.03, 0.06 0.12, 0.25, 0.5 and 1 pfu/nl) yield cell surface receptor expression levels of approximately 0, 3000, 7000, 14000, 25000, 50000 and 85000 mGnRHR/cell, as compared to physiological GnRHR levels that have been estimated at 20000–80000 sites/cell in pituitary gonadotropes and gonadotroph-lineage cell lines [52], [53]. Binning data according to ppERK2-GFP level revealed a clear positive relationship between ppERK2-GFP bin centre and ERK2-GFP N:C in cells treated with 0.25 pfu/nl Ad mGnRHR and treated for 15 or 30 min with GnRH, whereas no such relationship was seen in control cells without GnRH (Fig. 10C). Similarly, Ad mGnRHR titre-dependence calculated for a single ppERK2-GFP bin (120–160 AFU) and for cells stimulated for 15 min with GnRH revealed a clear TEY phosphorylation unattributable ERK2-GFP translocation (Fig. 10D) at titres that yield sub-physiological GnRHR expression levels (0.03–0.125 pfu/nl) as well as titres giving physiological expression levels (0.25 and 0.5 pfu/nl).

## Discussion

Specific biological responses to ERK pathway activation are partly achieved through the dynamic control of ERK subcellular localization [54]. This restricts the vast array of substrates available to ERK and enables phosphorylation of appropriate protein subsets. The translocation of ERK to the nucleus is a hugely important step in the transmission of extracellular signals to the genome. ERK does not contain nuclear localization or export sequences, and there is no consensus over a dominant mechanism for its traffic across the nuclear envelope, suggesting there is a high degree of redundancy in this step [6]. In resting cells, ERK is characteristically cytoplasmic due to its association with MEK and a number of other cytoplasmic anchors [6], [55]. Dual phosphorylation of the TEY motif causes the adoption of an active conformation, the dissociation of ERK from cytoplasmic anchors (exposing previously masked binding sites) and structural changes in docking motifs. These alterations enable ERK to interact with nuclear pore complex proteins, which facilitate passage to the nucleus [22], [56], [57].

Whilst MEK is of crucial importance for cytoplasmic retention of ERK in unstimulated cells, TEY phosphorylation of ERK can be uncoupled from nuclear localization by a variety of mechanisms. Striking examples of this are when 7TM receptors utilise β-arrestin scaffolds to signal to restrict phosphorylated ERK to endosomal complexes in the cytoplasm [27], or through neosynthesis of phosphatases capable of both dephosphorylating and anchoring ERK in the nucleus [36], [37], [43]. However, the first of these mechanisms is unlikely to be pertinent to mammalian GnRHR signalling because these receptors do not bind arrestins and therefore do not undergo the switch from G-protein-mediated to arrestin-mediated arrestin activations seen with other 7TM receptors [2]. The second mechanism is also unlikely to influence rapid GnRH effects because, although GnRH increases expression of DUSP1, 2, 4 and 5 mRNAs these effects are not seen until at least 15 min after GnRHR activation [44], [45].

We have optimised methods to explore relationships between ERK activation and location using high throughput microscopy to quantify TEY phosphorylated ERK (ppERK) and ERK nucleo-cytoplasmic distribution (N:C ERK ratio) in the same cells. Our previous work using data binning of single cells to compare ERK localization at matched whole cell ppERK levels revealed a previously unidentified component of the ERK nuclear accumulation response. Both PKC and EGFR activation induced an increase in ERK nuclear accumulation, even under conditions of identical TEY phosphorylation. This uncoupling seen at early time-points of stimulus was uninfluenced by protein synthesis or tyrosine phosphatase inhibition, suggesting that the uncoupling reflects post-translational modifications mediated by stimulus rather than differences in down-stream termination mechanisms [24]. Since GnRH-stimulated ERK activation is largely mediated by EGF receptor trans-activation and/or PKC activation [2], our primary aim in the present study was to determine whether the GnRHR also engages this TEY phosphorylation unattributable ERK translocation response. Our most important finding is that it does indeed do so, causing a rapid, transient and dose-dependent increase in N:C ERK, as compared to control cells, even under conditions where ppERK levels do not differ between control and stimulated cells. To our knowledge, this is the first demonstration that 7TM receptor activation can induce ppERK unattributable ERK redistribution to the nucleus. The kinetics of the response may be particularly pertinent to GnRH signalling, as the peptide is secreted in pulses with duration of only a few minutes [1]. Here, it is important to note that the uncoupling of TEY phosphorylation from translocation occurred at a broad range of receptor expression that encompasses endogenous GnRHR levels. Accordingly, this additional process increases the efficiency of nuclear ERK translocation in a physiologically relevant time-scale and at physiologically relevant receptor expression levels, and could therefore increase the efficiency with which GnRHR-mediated ERK activation affects nuclear targets with physiological (pulsatile) activation.

We used pharmacological inhibitors of EGFR and PKC activity to explore the mechanisms underlying GnRH-induced changes in ERK localization that are unattributable to TEY phosphorylation. We found that PKC inhibition only partially inhibited GnRH induced ppERK levels in population averages, but that it had more pronounced effects on GnRH induced nuclear localization of ERK. Examination of single cell data confirmed that PKC inhibition reduced nuclear translocation of ERK even at the same ppERK levels as in control conditions, indicating that PKC activation by GnRH facilitates nuclear localization of ERK via mechanisms additional to increasing TEY phosphorylation. These data are consistent with previous studies in this system and in LβT2 cells [31], [46]. Choi and colleagues demonstrated that GnRH stimulation of LβT2 cells causes PKC-dependent phosphorylation of PEA15, which is a cytoplasmic ERK anchor [46]. This causes the release of ERK from PEA15 and increases its availability for nuclear translocation. PEA15 can associate with both non-phosphorylated and TEY phosphorylated ERK. Therefore, if PEA15 plays a large role in PKC-dependent nuclear localization of ERK, TEY phosphorylation should be necessary but not sufficient for the full ERK nuclear localization response. Consistent with this model, our data indicate that phosphorylation of the TEY motif by MEK is an absolute requirement for nuclear localization of ERK induced by GnRH and other stimuli. Our data also show that the PKC-dependent component of the GnRH-induced response is chiefly responsible for nuclear translocation, and that this requires signals that are additional to TEY phosphorylation in a HeLa cell model.

Our previous work revealed that D319N mutation of ERK2-GFP abrogated the TEY phosphorylation-unattributable nuclear translocation response [24]. The data presented here show that this is also the case for GnRH. Reduction of D-domain binding (using the D319N mutation) had no measurable effect on ERK phosphorylation stimulated by GnRH and marginally reduced the average levels of nuclear accumulation, but greatly reduced the phosphorylation unattributable component of the nuclear localization response. PKC inhibition did not have an additive effect when used in combination with the D319N mutation. This suggests that PKC activity acts upstream of D-domain protein binding during TEY unattributable nuclear accumulation. This is consistent with a model where PKC activation mediates ERK activation and also facilitates ERK nuclear translocation through a separate route requiring engagement of D-domain containing proteins but is apparently independent of protein neosynthesis, ERK catalysis or engagement of ERK with DEF-domain containing proteins.

Taken together, we show that GnRH-induced phosphorylation of ERK by MEK on the TEY motif is necessary but not sufficient for GnRH to cause the full nuclear localization response. We find that there is an additional TEY phosphorylation unattributable component of the ERK translocation response that is provoked by GnRH (as well as by PKC or EGFR activation) and does not reflect induced expression of nuclear anchors. This component is reliant on PKC activation and binding of ERK to stimulus-regulated D-domain containing proteins. These studies highlight how GnRH induced signalling to ERK may be selectively modulated through altering subcellular targeting of ERK without necessarily affecting catalytic activity, which may represent more selective ways of controlling ERK behaviour in GnRHR-dependent pathologies.
